# Anti-arthritic efficacy of *Bombax ceiba ethanolic* extract in a murine model for rheumatoid arthritis using *in vivo*, *in vitro* and radiological analysis

**DOI:** 10.6026/97320630019833

**Published:** 2023-08-31

**Authors:** Bhargavi Posinasetty, Radhika Chikatipalli, Santenna Chenchula, Anitha Kuttiappan, Swapna G, Lakshmi G.N.A., Nelavala Audinarayana

**Affiliations:** Department of Clinical Data Management, Prometrika LLC, Cambridge - MA 02140, United States; Department of Pharmacology, Sri Venkateswara College of Pharmacy, Chittoor - 517127, A.P., India; Department of clinical pharmacology, All India Institute of Medical Sciences, Bhopal, India, 462020; Amity Institute of Pharmacy, Amity University Madhya Pradesh (AUMP), Gwalior - 474005, Madhya Pradesh, India

**Keywords:** *Bombax ceiba*, Freund's complete adjuvant-induced arthritis, haematological, histopathological and organ wt.

## Abstract

Rheumatoid arthritis (RA) is a multisystem autoimmune disease that causes discomfort, synovial membrane inflammation, peripheral joint inflammation, morning stiffness, articular tissue loss, and restricted joint movement. In the present study, we aim to explore the anti-arthritic efficacy of *Bombax ceiba* ethanolic extract in a Freund's Complete Adjuvant-induced arthritis, in murine model. The hot soxhlet method was used to extract dried aerial components of *Bombax ceiba * using an ethyl alcohol: water (70:30) ratio. *Bombax ceiba* ethanolic extract at two doses of 200 and 400 mg/kg was investigated in Wistar rats against Freund's full adjuvant-induced chronic immunological arthritis. Anti-arthritis efficacy was studied utilising morphological research (paw volume, paw diameter, and body weight). On the 28th day, the animals were sacrificed, and haematological parameters, pro-inflammatory cytokines (TNF-alpha, IL-6), cell culture, histological and radiological analysis were performed. BCEE inhibited paw oedema significantly (P 0.05) at a dose of 40mg/Kg, which was corroborated by paw volume and diameter, as well as haematological parameters, in Freund's complete adjuvant-induced arthritis model. The BCEE also significantly reduced pro-inflammatory cytokine levels and the histopathological changes caused by Freund's full adjuvant model. BCEE preserves synovial membranes by enhancing health and has shown a significant anti-arthritic activity. Thus, data confirms the traditional usage of *Bombax ceiba* for arthritis.

## Background:

Rheumatoid arthritis (RA) is a systemic, chronic, progressive inflammatory autoimmune condition that manifests as symmetric polyarthritis with swelling and pain in several joints, most commonly in the hands and feet.The joint is the primary site of inflammatory tissue destruction in RA, which is a complicated, multi-system disease. RA is a multisystem autoimmune disease that causes discomfort, synovial membrane inflammation, peripheral joint inflammation, morning stiffness, articular tissue loss, and restricted joint movement [1]. RA pathophysiology and aetiology are complicated and unknown. Destructive alterations in cartilage and bone, as well as bone outgrowths, limit joint motion. Arthritis can cause severe disability and ultimately affects a person's ability to carry out everyday tasks, restricts the quality of life and causes premature death [2].RA is the most common inflammatory disorder affecting about 1% of the global adult population; females are three times more prone to RA than males [3]. Traditional RA treatment with NSAID_s_, corticosteroids, immune suppressants, and anti-rheumatic medicines (TNF-alpha and monoclonal antibodies) has several limitations [4]. Chronic use of the aforementioned medications causes major side effects such as GIT ulcers, and cardiovascular, haematological, and renal toxicity [5]. Patients with persistent autoimmune illnesses are encouraged to seek alternative symptomatic alleviation [6]. *Bombax ceiba* Linnaeus is a member of the Bombaceae family [7]. It is widely found in temperate Asia, Tropical Asia, Africa and Australia [8].Many components of the plant (root, stem bark, gum, leaf, prickles, flower, fruit, seed, and heartwood) are used to treat a range of diseases by tribal societies and forest dwellers [9]. In the review of published literature on the traditional uses, biological activities, and isolated compounds from *Bombax ceiba * [10], pharmacological studies confirmed that the crude extracts or individual compounds from the plant showed Anti-diabetic activity, antimicrobial activity, anti urolithiatic activity, anti-inflammatory activities, hepato protective activity, and anti-hyperglycemic activity. Its stem bark extract can lower blood pressure [11]. Stem bark extract has antibacterial and antioxidant properties [12]. Therefore, it is of interest to document the arthritic efficacy of *Bombax ceiba ethanolic* extract in a murine model for rheumatoid arthritis using *in vivo*, *in vitro* and radiological analysis.

## Materials and Methods:

## Plant collection and authentication:

**Figure 1:**
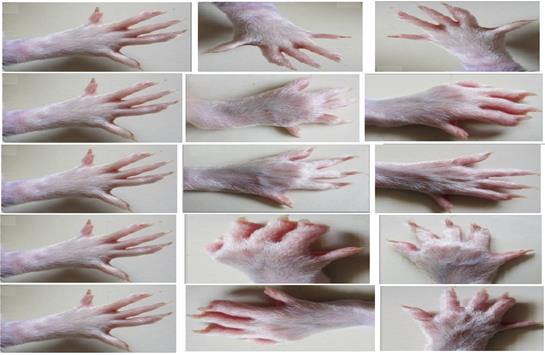
Photographic analysis (changes in paw diameter) of CFA induced arthritis in Wister rats.

The *Bombax ceiba * plantswere purchased as leafy vegetables from the local market of Pileru, Chittoor District, Andhra Pradesh state of India in March 2017; the photograph of which is shown in (Figure 1). Authenticationof the plant was carried out by Dr. K. Madava Chetty, Asst. Professor, Dept. of Botany, Shri Venkateswara University, Tirupati, A.P., India. Avoucher specimen of theplant (Ref. No. 0610 dated 11/09/2017); has been preserved there forfuture reference.

## Extract preparation:

The aerial plants were obtained by cutting the root sections, carefully washing them with tap water, air drying them in the shade, powdering them in a grinder, and passing them through Sieve No. 40 (ASTM). The dry powder was defatted with petroleum ether before being extracted with ethyl alcohol: water in a (70:30) ratio using the hot Soxhlet technique.The ethanolic extract was concentrated under reduced pressure in a rotary evaporator (Heidolph Instrument, Laborota 4000, Germany). The dried crude *Bombax ceiba ethanolic* extract (BCEE) was collected and stored at 4-8 °C in an airtight glass jar until use.

## Experimental animals:

The experiments were carried out on male Wistar rats weighing between 180 and 200 g. Sree Venkateshwara Enterprises in Bangalore, India provided all of the animals. The Institutional Animal Ethics Committee of Sree Vidyanikethan College of Pharmacy, Tirupati, Chittoor Dist., A.P., India (Approval No.: SVCP/IAEC/I-001/2018-19 dated 01/04/2019) approved all animal experiment protocols following the guidelines of the Committee for the Purpose of Control and Supervision of Experiments on Animals (CPCSEA). The animals were housed in Polypropylene cages and kept at 24°C 2°C on a 12-hour light/dark cycle, fed a normal pellet diet, and had free access to water.

## Experimental design:

A total of 30 male albino Wistar rats weighing 180-200 g were chosen and divided into five groups of six rats each (n = 6). Group I served as the standard control. Group 2 was utilized for arthritis control, Group 3 for Diclofenac 10 mg/kg, Group 4 for treatment group BCEE 200 mg/kg, and Group 5 for treatment group BCEE 400 mg/kg

## Dose selection:

Acute toxicity studies on the BCEE were not done because its safety of up to 200 mg/kg had already been documented [13]. Based on previous research, the two dosages of 200 and 400 mg/kg were chosen.

## Evaluation of anti-arthritic activity:

## Freund's complete adjuvant-induced arthritic rats:

Freund's complete adjuvant (FCA) comprises 10 mg/mL of heat-killed dead Mycobacterium tuberculosis bacteria in liquid paraffin [14]. Except for the rats in the normal control group, all rats were injected intradermally with 0.1 mL of FCA into the left hind paw on day '0'.The development of arthritis was given a 7-day interval. During this time, all of the animals acquired arthritic symptoms such as swelling, redness, and restricted movement [15]. The treatment came to an end on day 28.

## Morphological studies:

## Paw volume and diameter studies:

Rat paw volume and thickness were measured once every 7 days from the day '0' to '28' using a Plethysmometer (UGO Basile, Italy 7140) and a vernier calliper.

## Body wt. studies:

Body weight was assessed before and after induction using an electronic balance (Shimadzu C054-E032S, Japan), and the mean difference in body weight was recorded.

## Haematological studies:

On the 28th day, 3 mL of blood was obtained through retro-orbital puncture for the estimation of serum parameters (SGOT, SGPT, ALP, and Total protein) and blood parameters (ESR and%HB) using various diagnostic kits and conventional protocols. Later, on the 28th day, rats were euthanized with a strong dosage of halothane, and the ankle joints were used for additional radiological, histological, and organ weight examinations.

## Histopathological studies:

The ankle joints were removed on the 28th day and fixed in 10% buffered formalin. The bones were decalcified in 5% formic acid, paraffin-embedded, sectioned at 5 m thickness, and stained with haemotoxylin-eosin before being examined under a light microscope for changes in the synovium, cartilage, and joint space.

## Organs wt. studies:

On the 28thday, the spleen and thymus were removed, and the weight of the organs was recorded and corrected for 100 g body wt [16].

## Measurement of pro-inflammatory cytokines (TNF-alpha and IL-6):

The serum was isolated from the blood of experimental animals by clotting at room temperature for 30 minutes. TNF-alpha and IL-6 protein concentrations in serum were measured using ELISA kits and the method was carried out according to the manufacturer's instructions [17].

## Cell Culture:

RAW 264.7 cells were cultivated in 100 units/ml penicillin, 100 g/ml streptomycin, Foetal bovine serum, and 10% heat-inactivated at 37 ° C with 5% CO2. Cells were washed and detached using 0.25 percent EDTA-Trypsin in DMEM medium. The cells were re-suspended at a density of 2 106 cells/ml in a DMEM medium.

##  MTT (3-(4, 5-dimethylthiazolyl-2)-2, 5-diphenyltetrazolium bromide) assay:

The RAW 264.7 murine macrophage cell line was plated into a 96-well plate at a density of 1 104 in 100 L of DMEM media per well and incubated overnight until confluence was reached for treatment the next day. During treatment, the media was changed with different concentrations of plant extracts, including 12.5, 25, 50, 100, 200, 300, and 400 g/mL, and the cells were cultured for another 24 hours. After 24 hours of treatment incubation, 10 L of 5 mg/mL MTT solution was applied. The plate was centrifuged at that time, and the solution was removed, leaving the crystal in the bottom of the plate. The insoluble formazan salt was then dissolved with 100 L dimethyl sulfoxide [18]. The dish was gently stirred and placed in a dark room for about 30 minutes. The formazan produced was quantified using a micro plate reader with an absorbance value of 570nm.

## Radiological changes:

Lower limb X-rays were collected using a Siemens Heliphos D X-ray machine, and joint alterations were measured using joint space and soft tissue swelling [19].

## Statistical analysis:

The results were presented as Mean SEM (n = 6).Thestatistical significance was determined using the student t-test or one-way analysis of variance (ANOVA) followed by Dunnet's test, with P0.05, P0.01, and P0.001 considered statistically significant.

## Results and Discussion:

Freund's complete adjuvant-induced arthritic rats:

## Morphological studies:

## Paw volume and diameter studies:

Table 1 shows the effect of BCEE on paw volume and diameter in FCA-induced arthritic rats. The challenge with CFA (0.1 mL) results in the development of paw edema, which reaches a peak on the 21st day of injection. On days 7th (P0.05), 14th (P0.01), 21st (P0.001), and 28th (P0.001), the diclofenac-treated group significantly inhibits paw edema. BCEE (200mg/kg) inhibits paw edema significantly on days 21 and 28 with (P0.01). Furthermore, rats treated with BCEE (400 mg/kg) showed significant suppression of paw edema on days 7 (P0.05), 14 (P0.05), 21 (P0.01), and 28 (P0.01). Paw diameter grew until the 21st day of adjuvant induction when it slightly reduced. The diclofenac-treated group has significantly reduced paw diameter on days 14 (P0.01), 21 (P0.001), and 28 (P0.001). BCEE (200 mg/kg) inhibits paw diameter significantly on days 21 and 28 with (P0.01). Furthermore, rats treated with BCEE (400 mg/kg) showed significant inhibition in paw diameter on days 21 and 28 (P0.01). (Figure 1) depicts a morphological examination of rat paws.

On days 7th (p≤0.05), 14th (p≤0.05), 21st (p≤0.01), and 28th (p≤0.01), rats administered with BCEE (40 mg/kg) showed significant paw edema suppression. The paw diameter increased and then dropped somewhat until the 21st day of adjuvant induction. On day 14 (p0.01), 21 (p0.001) and 28 (p0.001) the diclofenac group revealed significant paw diameter inhibition. BCEE (200 mg/kg) inhibits paw diameter significantly on days 21 and 28 with (p0.01). Furthermore, rats treated with BCEE (400 mg/kg) showed a substantial inhibition in paw diameter on days 21 and 28 (p0.01). Table 1 shows the results. CFA-induced arthritic rats had considerably larger paw volumes than normal rats. When compared to arthritic rats, BCEE (low dose: 200mg/kg and high dose: 400 mg/kg b.w.) and diclofenac (10 mg/kg b.w.) treated arthritic rats demonstrated a substantial (p0.05) decrease in paw volume.

## Body weight studies:

Effect of BCEE on body weight in FCA-induced arthritic rats was tabulated in (Table 2) which indicates the increased body wt. during treatment of standard drugs and BCEE

## Haemato-logical studies:

Table 3 shows the effect of BCEE on various serum and blood parameters in FCA-induced arthritic rats. In the control group, CFA (0.1 mL) increases the levels of SGOT, SGPT, and ALP while decreasing the amount of total protein. The diclofenac-treated group has lower levels of SGOT (P0.01), SGPT (P0.01), ALP (P0.001), and higher levels of Total protein (P0.01). BCEE (20 mg/kg) has a substantial drop in SGPT (P0.05), ALP (P0.05), and an increase in Total protein (P0.05). The BCEE (400 mg/kg) treated group has lower levels of SGOT (P0.05), SGPT (P0.05), ALP (P0.01), and higher levels of Total protein (P0.05).

## Histopathological studies:

In the arthritis control group, histopathological studies of the rat ankle joint tissues (Figure 3) show destructive lesions in connective tissue, vascularity into joint space, and granuloma development. The ankle joint in the normal control group had normal connective tissue structure and no necrosis. Diclofenac treatment revealed normal connective tissue in the ankle joint, as well as decreased oedema and a lack of necrosis. By lowering inflammation and necrosis, BCEE-treated rats provided knee joint protection compared to arthritis control group rats. BCEE (200mg/kg)-treated rats developed granuloma, oedema, and necrosis, with little inflammatory cells. BCEE (400mg/kg) treatment resulted in mild necrosis with oedema, but no granuloma in the ankle joint.

## Organs weight studies:

The mean thymus weight decreased while the mean spleen weight increased in the FCA-treated animals compared to the NC group (Table 4). When compared to FCA-treated rats, the rise in spleen weight was considerably (P 0.01) suppressed by BCEE (20 and 40 mg/kg) and diclofenac (10 mg/kg). Only treatment with 400 mg/kg of BCEE plus diclofenac significantly reduced thymus weight loss (P 0.01).

## Pro-inflammatory cytokines (TNF-alpha and IL-6):

**Figure 2:**
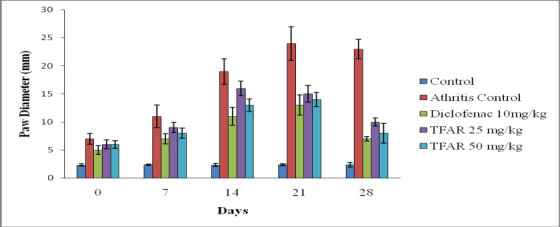
Effect of BCEE on Paw diameterin FCA-induced arthritic rats

**Figure 3:**
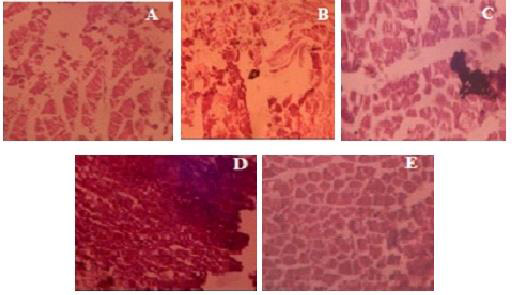
Histopathological observation of the rat ankle tissues (A) Normal control (B) Arthritis control(C) Diclofenac (10 mg/kg)(D) BCEE (200 mg/kg) (E) BCEE (400 mg/kg) treated rats. Magnification: x100; thickness: 5 µm.

**Figure 4:**
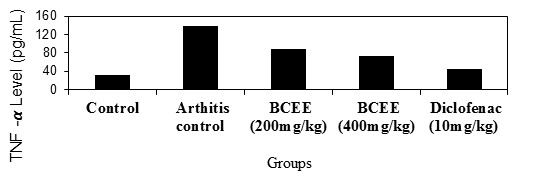
Effect of BCEE on TNF-Alpha in FCA-induced arthritis rats

**Figure 5:**
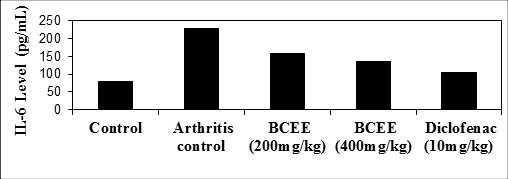
Effect of on IL6 level in FCA-induced arthritis rats

**Figure 6:**
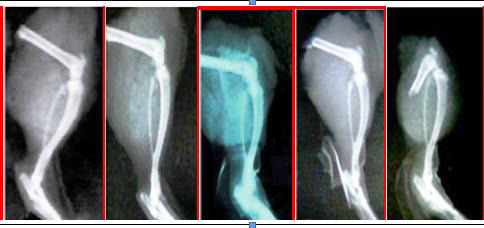
Radiographic analysis of CFA-induced arthritis in Wister rats. (A) Control group; (B) Negative control; (C) standard group; (D) BCEE (200mg/kg) ; (E) BCEE (400 mg/kg).

**Figure 7:**
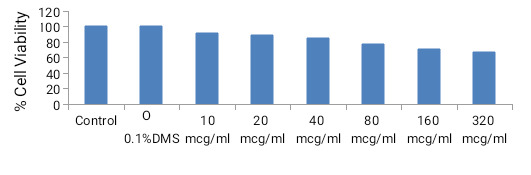
Effect of BCEE on the percentage of cell viability in RAW 264. 7 macrophages

BCEE (400mg/kg) demonstrated a significant (P0.5) effect compared to arthritis control in a study of proinflammatory cytokines. The results revealed that proinflammatory cytokine inhibition was dosage dependent. When compared to BCEE (400mg/kg) and the arthritis control, conventional diclofenac demonstrated a substantial (P0.01) reduction in proinflammatory cytokines. [Figure 2, Figure 4, Figure 5, Figure 6, Figure 7] depicts the results of TNF-Alpha and IL6 levels.

##  Radiographic study:

CFA-induced arthritic rats had visible tissue swelling, cystic expansion of bone, and widespread erosions, resulting in constriction or pseudo-widening of all joint areas. At the end of the BCEE therapy, rats administered with the usual medication diclofenac sodium showed reduced bone damage and produced arthritic animals.

In-vitro cell viability of previously cultivated RAW 264.7 cells was assessed in the presence of BCEE. The concentrations of BCEE were fixed at 10, 20, 40, 80, 160, and 320 g/mL. The results showed that BCEE at 160g/mL and 320g/mL significantly reduced RAW 264.7 viability to 71.23% 1.69% and 67.42%, 2.11%, respectively, as compared to the negative control. Meanwhile, BCEE at concentrations ranging from 10 to 80 g/mL was not harmful to RAW 264.7 cells since it did not reduce cell viability, but rather increased RAW 264.7 cell viability in comparison to the negative control. Our investigation confirmed that BCEE concentration affects cell viability by increasing or lowering it, which can be useful in determining the ideal dose.

## Discussion:

The most popular model is Freund's complete adjuvant (FCA) produced arthritis in rats. This preclinical model predicted the actions of a variety of drugs that are currently being investigated in clinical trials for the treatment of rheumatoid arthritis. The four phases of arthritis are based on biochemical parameters: Phase 1: Acute local inflammation of the liver and systemic effects (1-4 days); Phase 2: Remission of acute inflammation and periarthritis (7-12 days); Phase 3: Chronic inflammation, periarteritis, and osteogenic activity (12-28 days); and Phase 4: Permanent articular deformity and minimal inflammation (35 days and up). The purpose of this study was to determine the efficacy of a hydro-alcoholic extract of aerial portions of the indigenous herb *Bombax ceiba * against arthritis. Male Wistar strain rats were chosen for the current investigation because they develop persistent swelling in many joints due to the accumulation of inflammatory cells, erosion of joint cartilage, and bone deterioration. It closely resembles human rheumatoid illnesses [20]. The measurement of paw swelling appears to be a straightforward, sensitive, and rapid approach for assessing the level of inflammation and the therapeutic effects of medications. Freund's Adjuvant model was chosen because it causes chronic swelling in many joints due to the effect of inflammatory cells, resulting in joint cartilage erosion and bone deterioration. A variety of mediators, including cytokines (IL-1B and TNF-alpha), GM-CSF, interferons, and PGDF, are released during chronic inflammation. These mediators are responsible for pain, bone and cartilage degradation, and severe impairment [21].Standard drug and hydro-alcoholic *Bombax ceiba * extract, on the other hand, can significantly suppress paw swelling and decrease paw volume in both acute and chronic phases, which may be due to the suppression of inflammatory mediators released due to Freund's adjuvant induction [22]. Though the exact mechanism of inflammation suppression is unknown, it can be connected with the presence of alkaloids and flavonoids in suppressing inflammation and antioxidant activity [23]. Changes in the body weights of the rats occurred throughout the experimental period as the incidence and severity of arthritis increased. Previous research suggests that absorption of 14C-glucose and 14C-leucine in the intestine of inflamed rats is reduced [24]; however, when treated with anti-inflammatory drugs, the decrease in absorption is nullified, indicating that anti-inflammatory drugs correct the decreased/deranged absorption capacity of the intestine during inflammation [25]. The increased body weight during therapy with the usual medicine, hydro-alcoholic *Bombax ceiba * extract, could be attributed to the restoration of intestinal absorption ability. The extract has also been shown to have a considerable influence on a variety of blood and serum parameters [26]. Formaldehyde-induced arthritis is a typical acute paradigm for evaluating the anti-arthritic properties of plant extracts [27, 28,29]. Edema in the paw of the rat after injection of 0.1 mL of 2% v/v formaldehyde is caused by the production of histamine, serotonin, and prostaglandin-like chemicals at the injection site [30]. The substantial anti-inflammatory potential of BCEE may explain the inhibition of paw edema in formaldehyde-induced arthritis.

## Conclusion:

The results of the current study have shown that, the total flavonoid fraction (BCEE) of BCEE has shown to have promising anti-arthritic effects by lowering pro-inflammatory cytokine levels and maintaining spleen and thymus weight. We conclude that, BCEE can be used as a potential therapeutic agent to contro inflammation in both acute and chronic arthritic population.

## Figures and Tables

**Table 1:** Effect of BCEE on paw volume in FCA-induced arthritic rats

**Groups**	**Paw volume (mL)**				
	**Day 0**	**Day 7**	**Day 14**	**Day 21**	**Day 28**
Normal control	0.32±0.05	0.0.32±0.03	0.33±0.02	0.33±0.06	0.34±0.02
Arthritis control	0.36 ± 0.09	0.56 ± 0.09	1.12 ± 0.06	1.46 ± 0.04	2.09 ± 0.08
Diclofenac 10 mg/kg	0.37 ± 0.03	0.41± 0.21**	0.57±0.19**	0.43±0.21***	0.39±0.15***
BCEE 20 mg/kg	0.35 ± 0.02	0.48 ± 0.26	0.62 ± 0.16*	0.59 ± 0.12**	0.54 ± 0.12***
BCEE 40 mg/kg	0.36 ± 0.05	0.44± 0.17*	0.60 ± 0.20**	0.52 ± 0.21**	0.48 ± 0.23***
Values are expressed as mean ± SEM (n=6). *P<0.05,**P<0.01,***P<0.001 as compared with Arthritis control. (One-way ANOVA followed by Dunnet's test).

**Table 2:** Effect of BCEEon body wt. in FCA-induced arthritic rats

**Groups**	**Mean Body wt. (gm)**		**Mean Difference in Body wt**
	**Before Induction**	**After Induction**	
Values are expressed as mean ± SEM (n=6); *P<0.05, **P<0.01 as compared with control followed by Student's t-test.
Normal control	178±1.23	178±1.23	--
Arthritis control	168 ± 3.13	188 ± 2.4	12 ± 1.26
Diclofenac 10 mg/kg	176 ± 2.24	207 ± 1.38	19 ± 1.11**
BCEE200 mg/kg	174 ± 1.12	203 ± 3.21	17 ± 1.03*
BCEE400 mg/kg	178 ± 3.65	208 ± 5.01	13 ± 1.07*

**Table 3:** Effect of BCEE on haematological parameters in FCA-induced arthritic rats

Groups	**Serum parameters**				**Blood parameters**	
	**SGOT (IU/L)**	**SGPT (IU/L)**	**ALP (IU/L)**	**Total Protein (gm/dl)**	**ESR (mm/hr)**	**HB (gm %)**
Normal control	42.14±1.38	46.72 ± 1.70	139.21± 1.22	8.10 ± 0.09	12.54 ± 0.85	17.09 ± 0.66
Arthritis control	122.27±3.01	102.26 ± 1.87	262.36 ± 20.13	4.04 ± 0.22	39.11 ± 5.4	7.4 ± 0.6
Diclofenac 10mg/kg	51.42±2.55**	52.18±2.04**	141.65±8.27***	7.18± 0.20**	17.74±.5.04**	15.5±0.81**
BCEE200mg/kg	76 ±6.81	73.48 ±1.40*	196.81 ± 8.44*	5.84 ±0.47*	23.34 ± 3.12	11.2 ± 0.9**
BCEE400mg/kg	68.93 ± 4.31*	63.63 ± 4.45*	171.18 ± 9.68**	6.42 ± 0.31*	18.26 ± 2.31*	13.3±1.01**
Values are expressed as mean ± SEM (n=6). *P<0.05,**P<0.01,***P<0.001.as compared with control (One-way ANOVA followed by Dunnet's test).

**Table 4:** Effect of BCEE on thymus and spleen wtin FCA-induced arthritic rats

**Groups**	**Spleen wt.(mg/100 g b.wt.)**	**Thymus wt. (mg/100 g b.wt.)**
Normal control	189.53±3.12	100.5±1.01
Arthritis control	259.34±3.61	71.18±2.34
Diclofenac 10 mg/kg	199.83±4.20**	91.00±1.46**
BCEE 200 mg/kg	224.50±2.36**	83.50±1.43
BCEE 400 mg/kg	210.00±2.34**	85.75±1.53**
Values are expressed as the mean ± SEM (n= 6); *P<0.05, **P<0.01 as compared with control (One-way ANOVA followed by Dunnet's test).
